# Academic Output in Global Surgery after the Lancet Commission on Global Surgery: A Scoping Review

**DOI:** 10.1007/s00268-022-06640-8

**Published:** 2022-07-18

**Authors:** Zachary Fowler, Rohini Dutta, John L. Kilgallon, Adili Wobenjo, Soham Bandyopadhyay, Priyansh Shah, Samarvir Jain, Nakul P. Raykar, Nobhojit Roy

**Affiliations:** 1 Program in Global Surgery and Social Change, Department of Global Health and Social Medicine, Harvard Medical School, Boston, MA USA; 2 Department of Surgery, Zucker School of Medicine at Hofstra/Northwell, New Hyde Park, NY USA; 3 Christian Medical College and Hospital, Ludhiana, Punjab India; 4 WHO Collaborating Centre for Research in Surgical Care Delivery in Low-Middle-Income Countries, Mumbai, India; 5 Department of Neurosurgery, Brigham and Women’s Hospital, Boston, MA USA; 6 Department of Surgery, Kenyatta University, Nairobi, Kenya; 7 Nuffield Department of Surgical Sciences, Oxford University Global Surgery Group, University of Oxford, Oxford, UK; 8 Baroda Medical College, Vadodara, Gujarat India; 9 Dayanand Medical College and Hospital, Ludhiana, Punjab India; 10 Center for Surgery and Public Health, Brigham and Women’s Hospital, Boston, MA USA; 11 Division of Trauma, Emergency Surgery, Surgical Critical Care, Department of Surgery, Brigham and Women’s Hospital, Harvard Medical School, Boston, MA USA; 12 Department of Public Health Systems, Karolinska Institute, 171 77 Stockholm, Sweden; 13 The George Institute of Global Health, New Delhi, India; 14 Clinical Neurosciences, Clinical & Experimental Sciences, Faculty of Medicine, University of Southampton, Southampton, UK

## Abstract

**Background:**

The Lancet Commission on Global Surgery (LCoGS) published its seminal report in 2015, carving a niche for global surgery academia. Six years after the LCoGS, a scoping review was conducted to see how the term 'global surgery' is characterized by the literature and how it relates to LCoGS and its domains.

**Methods:**

PubMed was searched for publications between January 2015 and February 2021 that used the term ‘global surgery’ in the title, abstract, or key words or cited the LCoGS. Variables extracted included LCoGS domains, authorship metrics, geographic scope, and clinical specialty.

**Results:**

The search captured 938 articles that qualified for data extraction. Nearly 80% of first and last authors had high-income country affiliations. Africa was the most frequently investigated region, though many countries within the region were under-represented. The *World Journal of Surgery* was the most frequent journal, publishing 13.9% of all articles. General surgery, pediatric surgery, and neurosurgery were the most represented specialties. Of the LCoGS domains, healthcare delivery and management were the most studied, while economics and financing were the least studied.

**Conclusion:**

A lack of consensus on the definition of global surgery remains. Additional research is needed in economics and financing, while obstetrics and trauma are under-represented in literature using the term ‘global surgery’. Efforts in academic global surgery must give a voice to those carrying the global surgery agenda forward on the frontlines. Focusing on research capacity-building and encouraging contribution by local partners will lead to a stronger, more cohesive global surgery community.

**Supplementary Information:**

The online version contains supplementary material available at 10.1007/s00268-022-06640-8.

## Introduction

Surgical care was featured as a central component of Halfdan Mahler’s address to the World Health Assembly in 1979 on primary health care and health systems [1]. Unfortunately, the enthusiasm generated by this seminal address dissipated without traction for implementation among the global health community, which instead shifted focus to “less expensive” interventions ranging from oral rehydration therapy to immunizations [2, 3]. This would eventually lead to the famous description of surgery as the “neglected stepchild of global health” almost three decades later [4].

A series of critical milestones over the past fifteen years including the publication of The Lancet Commission on Global Surgery (LCoGS) renewed hope for the integration of global surgery into the global public health lexicon and health systems frameworks [5]. The LCoGS was particularly important because not only did it provide an overview of the current state of global surgery, but it also provided a framework for action split across the domains of health delivery, workforce, information systems, and economics.

Enthusiasm for surgical and anesthesia care in the global health arena may dissipate again, much as it did after Alma Ata, unless it is sustained by a broad range of clinicians, researchers, and policymakers, working in areas across the spectrum of health systems, and across high-income countries (HICs) and low- and middle-income countries (LMICs). Little is known, however, as to whether clinicians and researchers in LMICs working toward equity in surgical access for all populations identify with the phrase “global surgery” or the movement represented by the LCoGS. Previous studies have suggested an imbalance in authorship in global health research, with several bibliometric analyses finding that authors from LMICs are less likely to hold first and last author positions [6–8]. Some LMIC researchers have also expressed that the term ‘global surgery’ does not resonate with surgeons working in remote and rural areas [9, 10]. This raises the question of how the current academic output in global surgery aligns with the field as described by the LCoGS.

We aimed to characterize the use of the term ‘global surgery’ or citations of LCoGS in the global academic literature with an interest in quantifying the volume of literature produced, the health system domains to which it pertained, the geographic region it originates from and studies, and its distribution of authorship among HIC and LMIC researchers.

## Methods

We performed this scoping review in accordance with the framework by Joanna Briggs Institute and reported as per the PRISMA-ScR guidelines [11, 12].

### Data source

We searched publications from January 2015 to 7 February 2021 in the PubMed (MedLine) database. This database is widely used for reviews due to its expansive journal range, augmented citation analysis, and availability of non-Medline publications [13]. The literature search was limited from January 2015 until the day of performing the search to assess the body of literature published during and after the key global surgery events of 2015. No new studies were retrieved from other sources such as gray literature, non-peer-reviewed articles in newspapers and op-eds.

### Search strategy

We searched for articles containing the term “global surgery” in the title, abstract, or key words and articles citing the report of the LCoGS. All resulting articles were uploaded into the Covidence software (Covidence, Melbourne, Australia) for removal of duplicates, title and abstract screening, full-text review, and data extraction [14].

### Study selection

All study designs and all types of publications, including conference proceedings, editorials, commentaries, case reports and abstract-only texts were eligible for inclusion. Articles included were published in English and covered any aspect of global surgery.

### Study screening

Title and abstract screening and full text review were completed by four authors (ZF, RD, JK, AW). Two authors independently screened articles, and a third author resolved conflicts. If articles were not available for download, efforts were made to obtain them through ResearchGate, the Harvard University Countway Library and/or by contacting authors by email.

### Data extraction

Data were extracted by seven authors (ZF, RD, JK, AW, SB, PS, SJ). Data extracted by one author were verified by another for each article. Discrepancies were discussed, reviewed, and resolved by a third author. Data extracted are listed in Table 1. If an article reported more than one domain, all were noted.

**Table 1:** Variables extracted

Publication metrics
Journal
Year of publication
Scope (LMIC^A^/HIC)
*Authorship information*
Number of authors affiliated with an academic global surgery or global health program
Number of HIC and LMIC authors as per affiliations
Type of institutional affiliation
*Article type and methodology*
*Geographic scope*
African region (AFR)
The region of the Americas (AMR)
South-East Asia region (SEAR)
Western Pacific region (WPR)
Eastern mediterranean region (EMR)
European region (EUR)
*Surgical specialty*
General surgery
Pediatric surgery
Neurosurgery
Trauma surgery
Plastic surgery
Orthopedic surgery
Obstetrics and gynecology
Anesthesia
Surgical oncology
Cardiothoracic surgery
Ophthalmology
Otolaryngology
Urology
Dentistry
Others
*LCoGS Domain(s) investigated/reported*
Healthcare delivery and management
Workforce, training, and education
Economics and financing
Information management
*Other domains*
Academic global surgery programs
Clinical research
Surgical missions / volunteerism
Research capacity building
Global surgery partnerships
Global surgery ethics
Global health agendas (UHC, SDGs, WHA, etc.)

^*^A- The World Bank income group 2020 definition of an LMIC was used, which encompasses all countries whose gross national income per capita is less than US$12,375 (15)

### Data analysis

Microsoft Excel Version 16.57 was used to perform descriptive statistics.

## Results

**Fig. 1:**
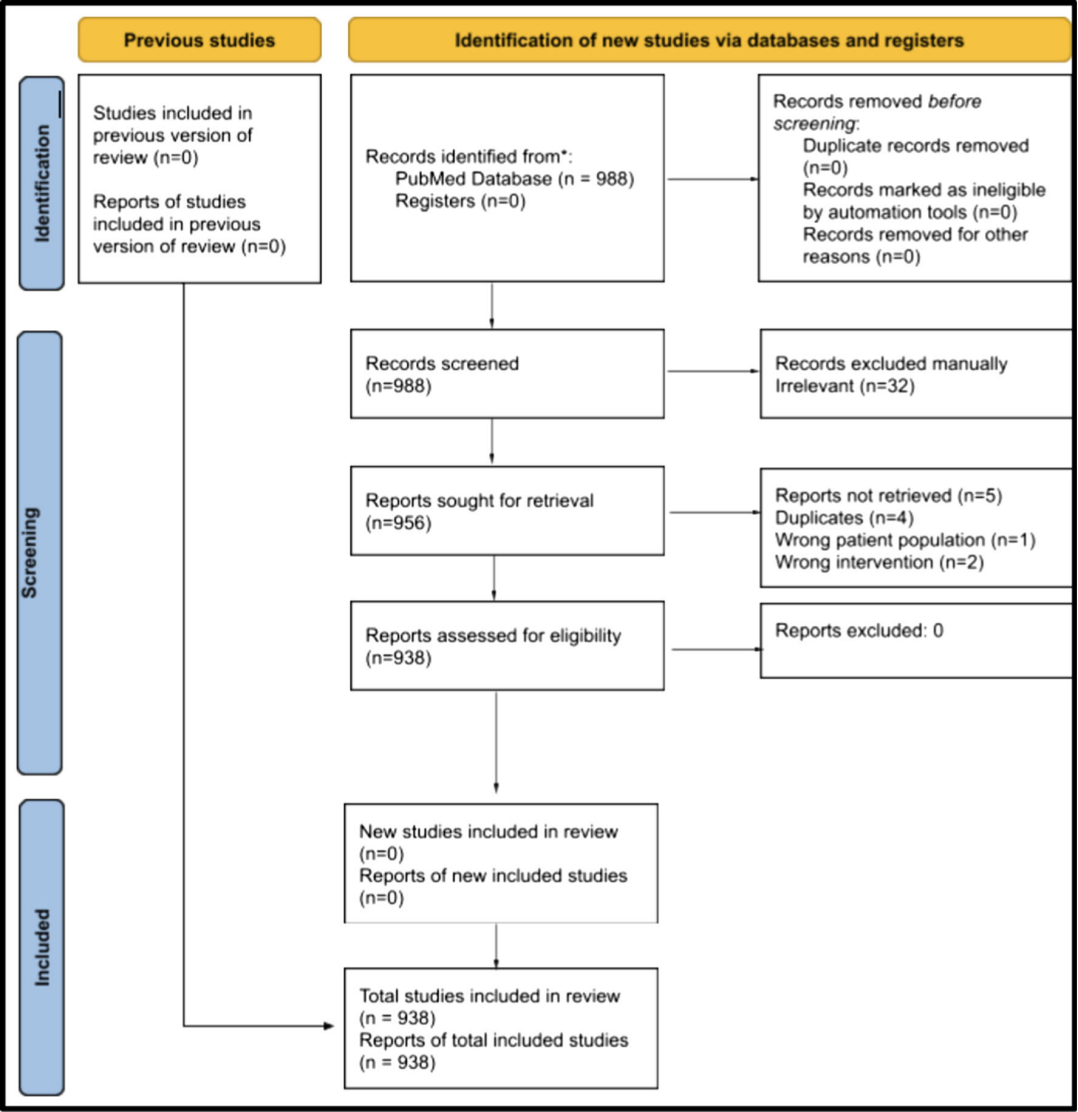
PRISMA scoping review flowchart

There were 988 articles in our initial search, all of whose titles and abstracts were screened for eligibility. Following this, 956 full texts were assessed, and 938 were eligible for inclusion in the data extraction phase (Fig. 1).

41.7% (*n* = 391) of the included articles cited the LCoGS, 23.9% (*n* = 224) had the phrase ‘global surgery’ in their title, abstract, or keywords, and the remaining 34.2% (*n* = 321) met both criteria. 53.3% (*n* = 500) of the articles were original research, while 17.6% (*n* = 165) were reviews and 29.1% (*n* = 273) were other types of articles, such as editorials, letters to the editor, commentaries, and proceedings. The *World Journal of Surgery* was the most frequent journal with 13.9% (*n* = 131) of all included articles. The top ten journals by the number of articles published are listed in Supplementary Material 1.

There were 6,453 authors among all included articles. 4,397 had HIC affiliations, 1,756 had LMIC affiliations, and 300 had both HIC and LMIC affiliations. The average proportion of HIC, LMIC, and both HIC and LMIC authors per article was 70.99%, 24.27%, and 4.74%, respectively. We found that 78.82% of the first authors and 78.26% of the last authors had HIC affiliations, whereas only 14.44% of the first authors and 15.15% of the last authors had LMIC affiliations. Authors who were affiliated with both HIC and LMIC institutions were first authors in 6.73% of the articles and last authors in 6.58% of the articles. A small percentage (2.3%) also used a collaborative research unit and published without naming specific authors. Among all articles, 40.2% had no authors with LMIC affiliations. We also found that the most common affiliation was HIC universities, present in 83.9% of articles. Frequencies of affiliation types are listed in Supplementary Material 2.

**Fig. 2:**
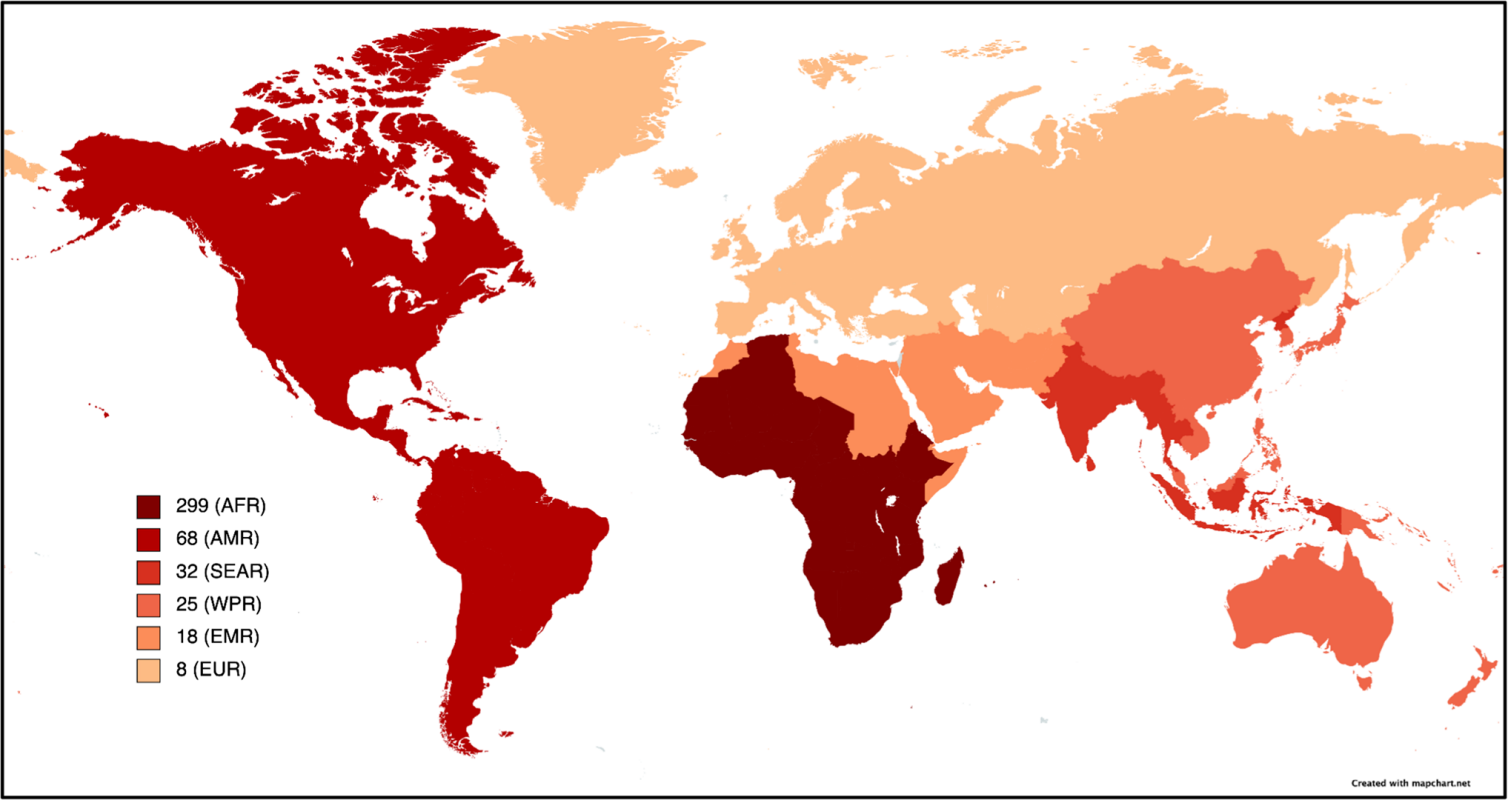
Number of articles with a target area within each WHO region (*n* = 449). Articles with a geographic focus on multiple regions or that did not list specific countries were not included

Articles varied in geographic scope; 44.14% (*n* = 414) had a global focus, 13.97% (*n* = 131) examined multiple countries, 26.55% (*n* = 249) focused on a single nation, and 15.25% (*n* = 143) studied a subnational region. All WHO regions were covered, and the number of articles with a target area within each region is shown in Fig. 2 [16]. When global studies were excluded, the African Region (AFR) was the most common WHO region studied at 57.06% [*n* = 299]. The Region of the Americas (AMR) was the second most common at 12.98% [*n* = 68], followed by South-East Asia Region (SEAR) at 6.11% (*n* = 32), Western Pacific Region (WPR) region at 4.77% (*n* = 25), Eastern Mediterranean Region (EMR) at 3.44% (*n* = 18), and European Region (EUR) at 1.53% (*n* = 8). 13.93% (*n* = 73) examined multiple regions or did not specify a region of focus. Of articles with a national or subnational scope (*n* = 392), the most common country of focus was Uganda at 9.69% (*n* = 38), followed by Rwanda at 7.91% (*n* = 31), Ghana at 7.65% (*n* = 30), Malawi at 7.40% (*n *= 29), and Tanzania at 5.36% (*n* = 21).

**Fig. 3:**
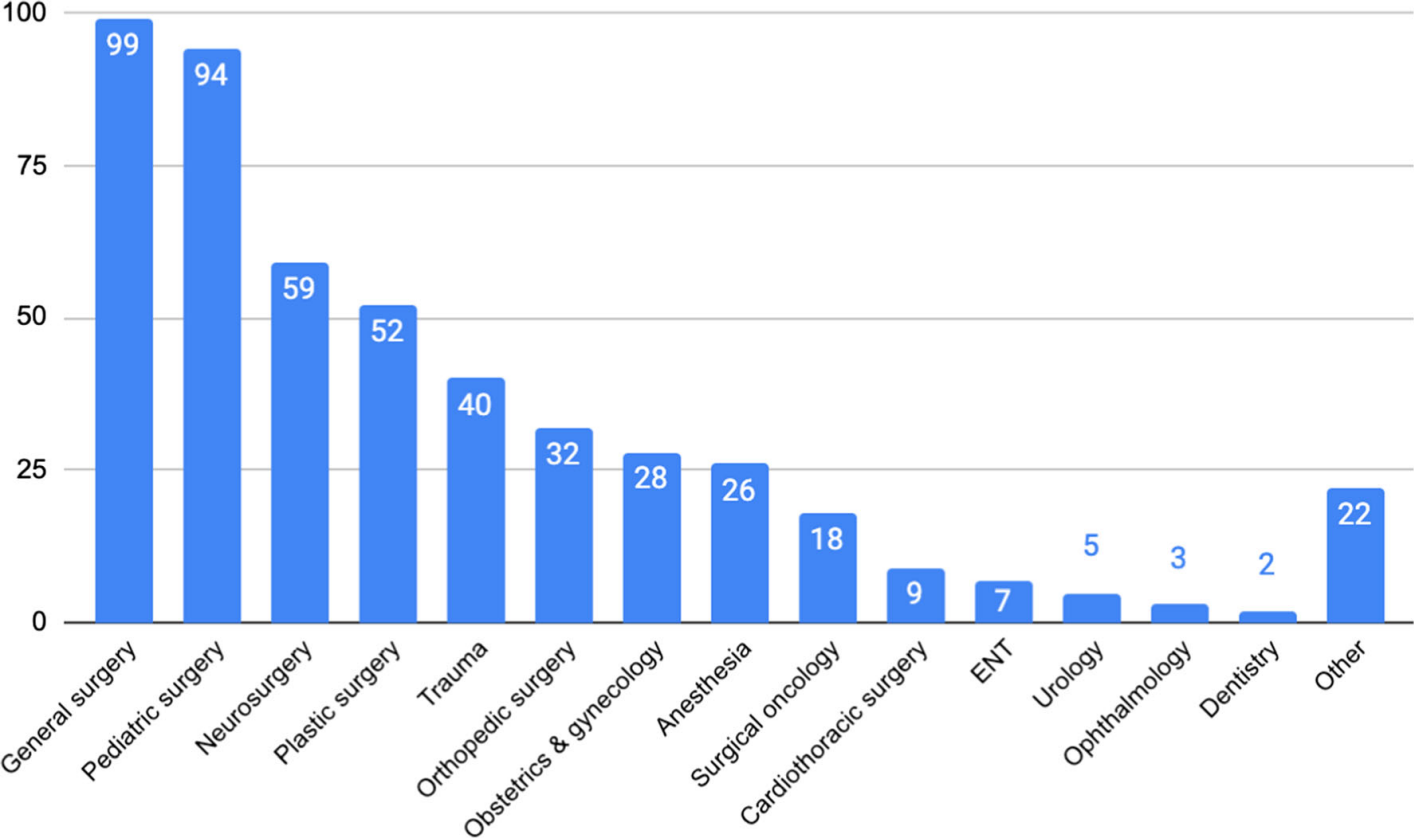
Number of articles by specialty

The most common specialties examined (excluding those that did not specify or noted multiple specialties) were general surgery (19.88%; *n* = 99), pediatric surgery (18.88%; *n* = 94), and neurosurgery (11.85%; *n* = 59). A list of specialties and number of articles are shown in Fig. 3.

**Fig. 4:**
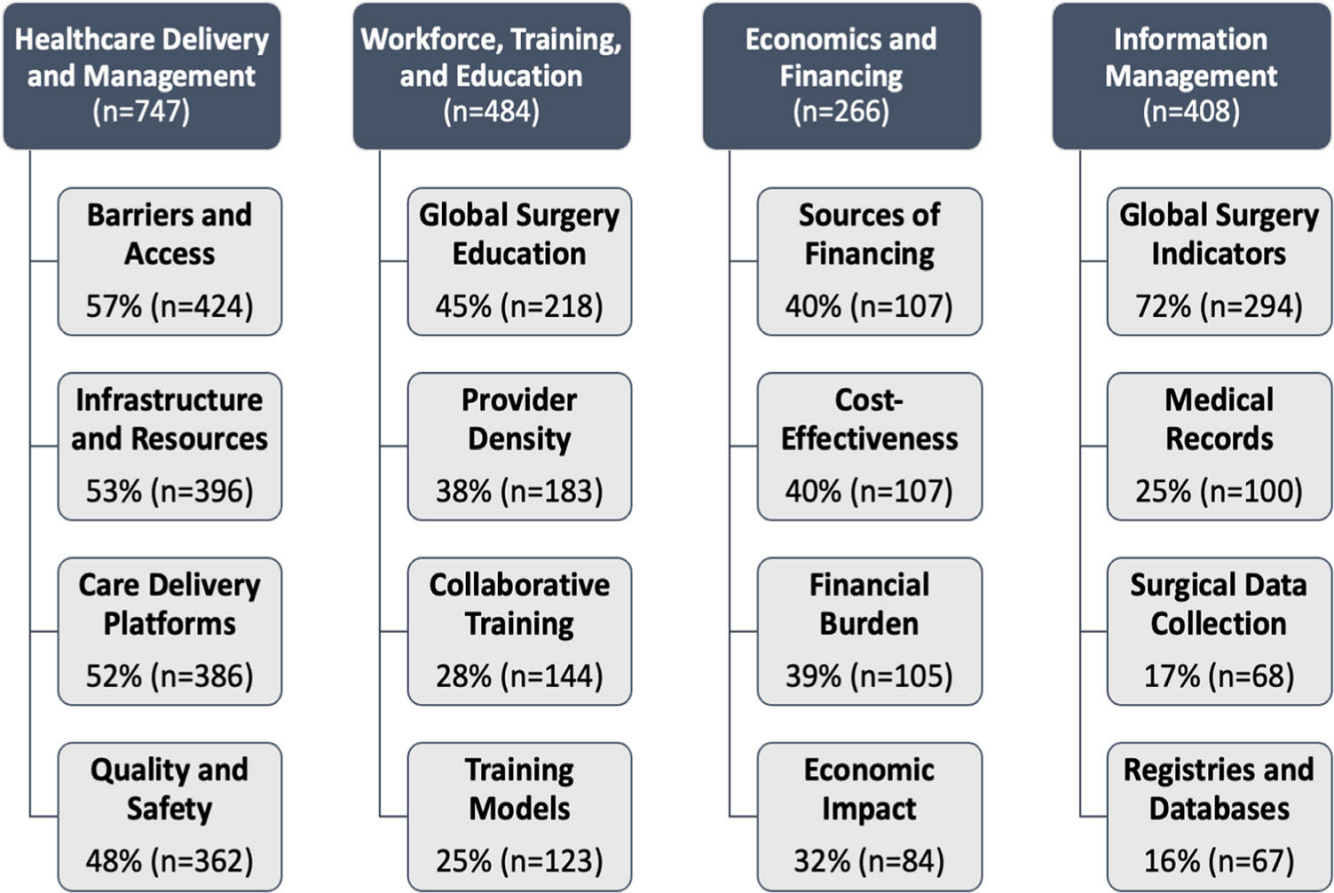
Frequency of sub-categories within primary LCoGS domains

Articles were categorized into the four primary LCoGS domains: (1) healthcare delivery and management (*n* = 747); (2) workforce, training, and education (*n* = 484); (3) economics and financing (*n* = 266); and (4) information management (*n* = 408) as shown in Fig. 4. The authors assigned one or more LCoGS domains being reported in the article and hence the number of articles per domain is not equal to the total number of articles.

In addition to these health system domains, other areas of study described by the LCoGS were assessed. While the bulk of the literature falls under the umbrella of health system strengthening, clinical research papers comprised 15.2% (*n* = 143) of the included studies. Surgical epidemiology was also frequently reported, with 37.6% of articles providing data (*n* = 353). The establishment of global surgery as an academic discipline is reflected in the number of papers with a focus on academic global surgery, with 16.1% (*n* = 151) of papers discussing the field within academic institutions.

## Discussion

In this assessment of global surgery literature after The Lancet Commission on Global Surgery (LCoGS), we found that 938 papers have been published using the term ‘global surgery’ in the abstract or title or have cited the LCoGS. These papers reflect research across the globe, spanning each WHO region, multiple transnational collaborations, and a broad range of clinical specialties. Further, they address all domains of health system strengthening including those outlined by the LCoGS: healthcare delivery and management, workforce and education, economics and financing, and information management.

A comparison with prior bibliometric analyses on global surgery suggests that the research output captured in this study does not encompass the full range of global surgery output being produced. Our results yielded a rough estimate of 156 articles being published per year since the establishment of the LCoGS in 2015. A 30-year bibliometric analysis from 1987 to 2017 by Sgro et al.identified 1,623 articles during which the number of annual publications increased from 14 to 149 [17]. Their search included articles about surgical research conducted only in LMICs and did not require the use of the term ‘global surgery’. Our search captured a similar number of articles in corresponding years but with differences in range and content, including country affiliations of authors and clinical specialties. A review by Pauyo et al.assessed surgery literature in low-income countries from 2002 to 2011 and included 2049 articles, the majority of which were case reports and case series [18]. Again, this review included a large number of articles from specialties that were less represented in our search, and the country affiliations of authors also differed. Based on these data, it appears likely that a significant amount of global surgery research in LMICs is not classified as ‘global surgery’ by its authors, nor seen as related to the LCoGS.

### Authorship trends

Among the articles included in this study, nearly 80% of all first and last authors had HIC affiliations, and 40.2% of articles had no LMIC authors. On the contrary, Sgro et al. found that more than two times as many authors had LMIC affiliations, and Pauyo et al.reported that more than half of included articles were authored solely by LMIC researchers. This could support the previous notion that the term “global surgery” and citation of the LCoGS is overrepresented among HIC researchers compared to LMIC researchers and that many LMIC researchers do not readily identify with “global surgery” or LCoGS. A review by Ravi et al.on authorship demographics in global surgery since 2016 found that 51% of authors were only affiliated with HIC institutions, which is consistent with our results [8]. Additionally, it is possible that there is a partial contribution to this imbalance of LMIC authors due to inequities in research participation between HIC and LMIC authors. While the issues of HIC and LMIC authorship are sometimes conflated with the concept of decolonizing global surgery, the arguments for equity in academic global surgery are more nuanced and complex. Despite many HIC researchers calling for and promoting equitable practices, various system-level factors may make the implementation of this challenging [19]. For example, incentive structures for grants, promotions, and tenure often require HIC academics to publish frequently and with first and last author positions [20]. On the contrary, researchers in LMICs tend to have less protected research time, weaker research infrastructure, and more demanding clinical duties [21]. Solutions should be directed at the academic environment to give more opportunities to LMIC researchers through capacity building, mentorship, and changes to academic incentives [22, 23]. Additionally, the value placed on first and last author positions may not be appropriate in global health research, which often involves many partners and large teams working together [24]. Academic institutions and societies involved in global health should define criteria and guidelines for meaningful co-authorship, adapting existing notions of fairness in academic authorship to the global surgery context [25–27]. Journals should also recognize and promote sharing of first and senior author positions through modifications in their policies and in submission portals.

### Geographic representation

While we found that all geographic areas were represented in the “global surgery” and LCoGS literature, several regions are less represented than others. The top five African countries comprised 38% of all articles with a national or subnational focus, but only 26 of the 47 African Region countries were represented in our results. Additionally, countries in the Eastern Mediterranean Region were the focus of only 3.4% of articles with a national or subnational focus despite 21 member states and a population of nearly 679 million [28]. This underrepresentation could reflect a persistent lack of self-identification as global surgeons among various LMIC surgical communities. Other terms such as “rural surgery” have been used to describe similar work in some areas, such as South Asia [9, 10]. This may result in a large portion of surgical research pertaining to low-resource and underserved communities from LMICs being classified out of the context of ‘global surgery’. Studies also tend to characterize and compare inequities at the level of the country or region, but large disparities within individual countries exist, may be less studied, or may again not be classified as “global surgery” or related to the LCoGS by authors when pertaining to domestic concerns.

### Clinical specialties and ‘global surgery’

When we examined studies in our results with a focus on a single surgical specialty, general surgery, pediatric surgery, and neurosurgery comprised over half of the studies. Trauma and obstetrics & gynecology were the subjects of only 8% and 6% of articles, respectively. Conversely, prior reviews have found that obstetrics and gynecology is one of the most represented specialties in global surgery literature [17, 18]. While the fields of obstetrics and trauma have very active global health research communities, much of their academic output was not captured by our search, suggesting again that researchers in these fields may not associate with the terms “global surgery” or the LCoGS [17, 18]. Meanwhile, pediatric surgery and neurosurgical organizations such as the Global Initiative for Children’s Surgery and the World Federation of Neurosurgical Societies have shown a high level of engagement with “global surgery” advocacy initiatives worldwide (29,30).

### Lancet commission on global surgery domains

LMICs are projected to lose $12.3 trillion USD between 2015 and 2030 due to the economic impact of surgical conditions [5]. We found that “economics and financing” was the least represented domain among our bibliometric search. Financing is a key component of National Surgical, Obstetric, and Anesthesia Plans, which have been completed in several LMICs [31]. National funding restraints and limited commitment from donors, however, have raised concerns about their implementation and impact [32, 33]. Ensuring durable funding mechanisms and understanding the impact of the cost of surgical care on patients will require further investigation and collaborative work including both clinicians and researchers.

This scoping review was meant to understand the growth and distribution of “global surgery” literature over the years since The Lancet Commission on Global Surgery and has several important limitations. We chose to track academic literature that used the term “global surgery” or cited the LCoGS but, as reflected in the discussion, these are not all-encompassing in capturing the true breadth of surgical research in LMICs pertaining to global surgery. Further, we searched a single database, PubMed, which may have excluded literature published in local journals not indexed in PubMed and excluded non-peer-reviewed articles including the gray literature and program reports, and our restriction to English-language articles excluded literature in non-English journals that may publish global surgery research in LMICs. Finally, the HIC or LMIC status of authors was derived based on their listed affiliation though we recognize their institutional affiliation (*e.g.*, international nongovernmental organizations) could not be reflective of the country in which they reside and work.

## Conclusion

A large volume of academic literature directly mentions “global surgery” or cites The Lancet Commission on Global Surgery, spanning a broad range of geographies, clinical specialties, and author affiliations between HICs and LMICs. However, a significant percentage of this work originates with authors from HICs, focused on specific areas of the world, and within certain clinical subspecialties. This suggests that a large proportion of research that pertains to global surgery may not identify with “global surgery” or The Lancet Commission on Global Surgery. Of studies that do mention “global surgery” or LCoGS, we found that research pertaining to the LCoGS domain of economics and financing is least represented. The *World Journal of Surgery* has led the dissemination of the global surgery agenda, and it may reflect the inclusive approach of this journal. The academic global surgery community must actively engage LMIC clinicians and researchers in “global surgery” efforts from across geographies and specialties and find innovative methods for supporting mutually beneficial partnerships.

## Supplementary Information

Below is the link to the electronic supplementary material.

Supplementary file1 (DOCX 94 KB)
