# Supplementary material for: Academic Output in Global Surgery after the Lancet Commission on Global Surgery: A Scoping Review
Source: World J Surg. 2022 Jul 18;46(10):2317–25. doi: 10.1007/s00268-022-06640-8 (PMC9436886; doi:10.1007/s00268-022-06640-8)
Supplement: Supplementary file 1 — Supplementary file1 (DOCX 94 KB) [file 268_2022_6640_MOESM1_ESM.docx]

**SUPPLEMENTARY MATERIALS**


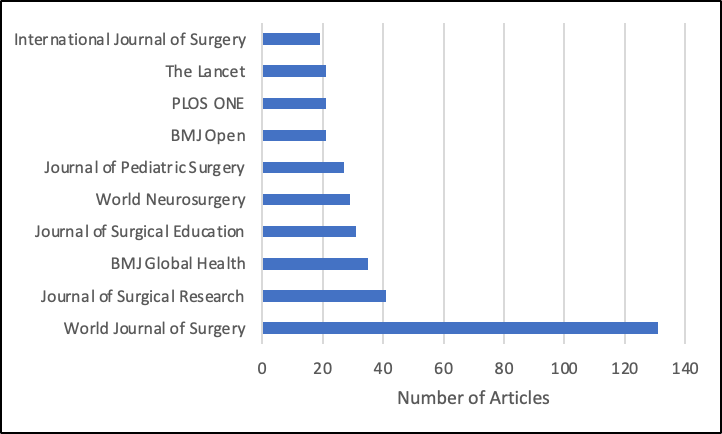


Supplementary file 1. Number of articles published by journal.


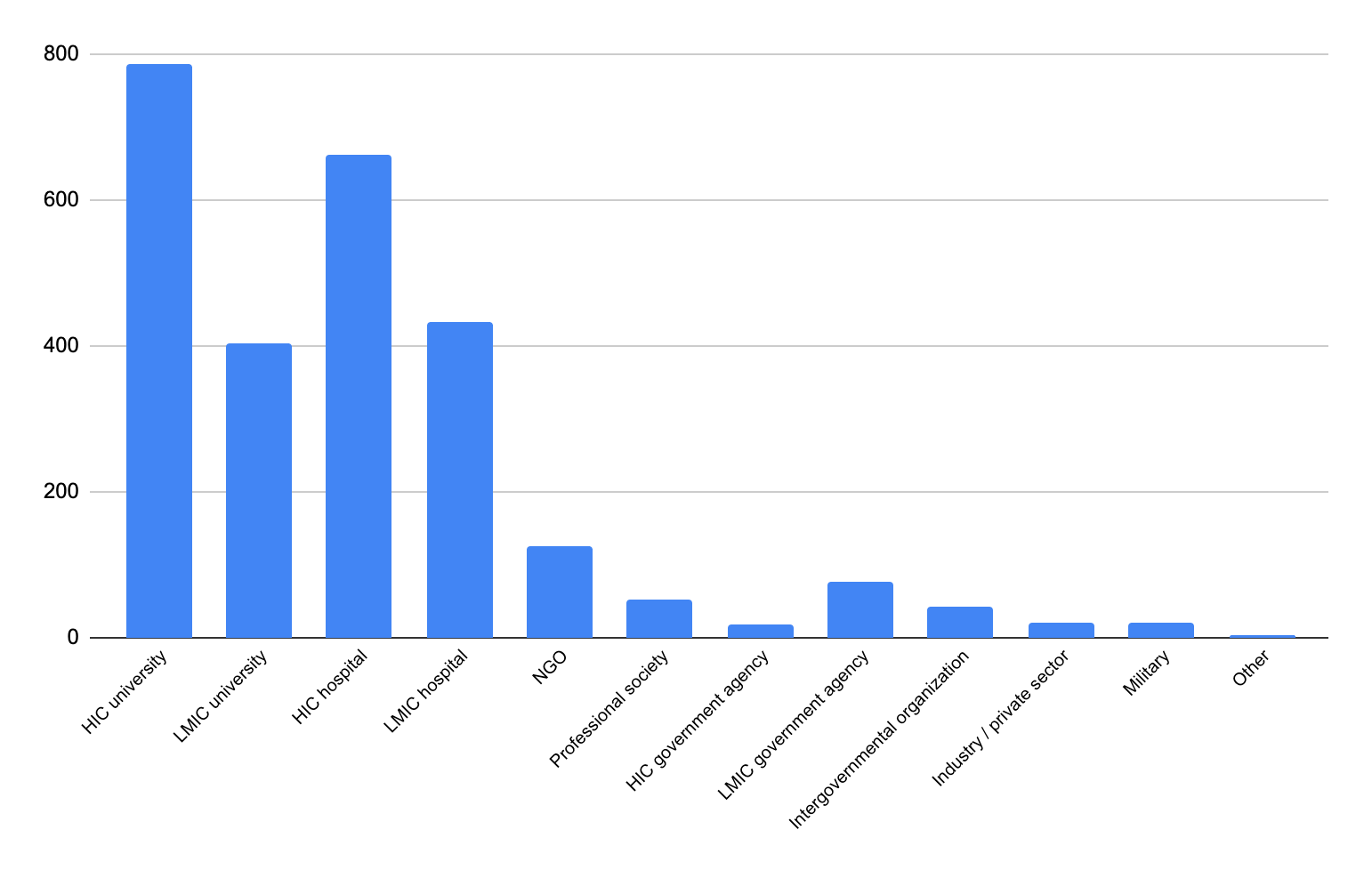


Supplementary file 2. Frequency of author affiliation category.
